# Successful management of an aortoesophageal fistula caused by a fish bone – case report and review of literature

**DOI:** 10.1186/1749-8090-4-21

**Published:** 2009-05-08

**Authors:** Stephen L Kelly, Paul Peters, Murray J Ogg, Alan Li, Bernard M Smithers

**Affiliations:** 1 Dept of Cardiothoracic Surgery, Princess Alexandra Hospital, Brisbane, Queensland, Australia; 2 Dept of Vascular Surgery, Princess Alexandra Hospital, Brisbane, Queensland, Australia; 3 Dept of Upper Gastro-intestinal and Soft Tissue Surgery, Princess Alexandra Hospital, Brisbane, Queensland, Australia; 4 University of Queensland, Princess Alexandra Hospital, Brisbane, Queensland, Australia

## Abstract

We report a case of aortoesophageal fistula (AEF) caused by a fish bone that had a successful outcome. Aortoesophageal fistula is a rare complication of foreign body ingestion from which few patients survive. Over one hundred cases of AEF secondary to foreign body ingestion have been documented but only seven, including our case, have survived over 12 months. Treatment involved stabilising the patient with a Sengstaken-Blakemore tube and insertion of a thoracic aortic endovascular stent-graft. Unfortunately the stent became infected and definitive open surgical repair involved removing the stent, replacing the aorta with a homograft and coverage with a left trapezius flap while under deep hypothermic arrest.

## Case presentation

A 59 year old man with no prior medical history, presented to a peripheral hospital emergency department with sharp pain retrosternally after eating fish the previous day. He was able to swallow fluids and soft diet but with odynophagia. A cardiac cause was ruled out and a barium swallow was organised as an outpatient. The patient was discharged home. Day five after presentation he had frank haematemesis and some malaena. He was haemodynamically stable with a haemoglobin at 130 g/L and admitted to the general surgical ward. The next day he had another large haematemesis and proceeded to urgent upper gastrointestinal (GI) endoscopy where a fish bone was seen protruding from an ulcerated area in the oesophagus, 24 cm from the teeth. There was some active bleeding after the fish bone was removed. A Sengstaken-Blakemore tube (SBT) was inserted and the oesophageal and gastric balloons inflated. This controlled the bleeding and the patient was admitted to the intensive care unit (ICU) with intravenous (IV) antibiotics as well as an ongoing blood transfusion. Overnight there was no further bleeding but the patient had evidence of sepsis with a high fever and hypotension despite no evidence of blood loss. He required inotropic support.

**Figure 1:**
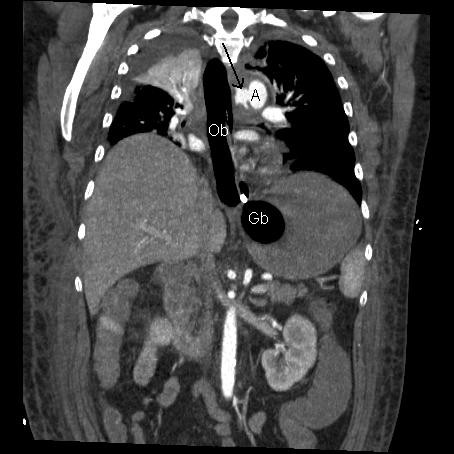
**CT angiogram – coronal view**. The oesophageal balloon (Ob) and gastric balloon (Gb) from the Sengstaken-Blakemore tube are easily visible. The black arrow points to the leakage of contrast from the aorta (A) indicating a breach in the aortic wall.

**Figure 2:**
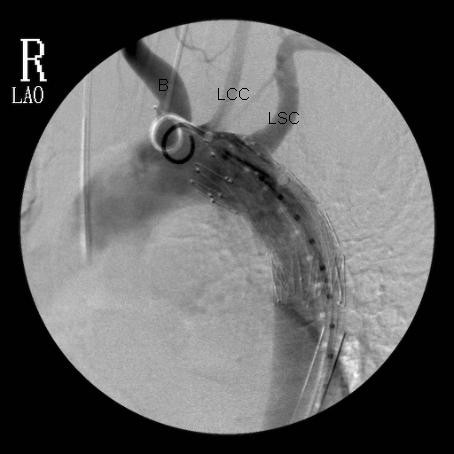
**Arch Aortogram showing the endovascular stent-graft**. The brachiocephalic artery (B), left common carotid artery (LCC) and the left subclavian artery (LSC) are labelled coming off the arch of aorta. The origin of the left subclavian artery has been covered, with some residual flow.

The following morning, the patient was transferred to the ICU at our centre. The SBT balloons were deflated and patient observed. There was no evidence of blood loss over the ensuing 24 hours so the SBT was removed. Five hours later, he had a massive haematemesis causing hypovolaemic shock with cardiac arrest requiring reinsertion and reinflation of the SBT, massive transfusion and cardiopulmonary resuscitation. Once stabilized, a computed tomography (CT) angiogram was performed. This demonstrated an aortoesophageal fistula located 2 cm distal to the origin of the left subclavian artery on the descending aorta (Fig 1). In the vascular intervention suite under image intensifier control, an endoluminal stent-graft (Zenith^® ^TX2™ Thoracic TAA Endovascular Graft – one piece Thoracic Body Extension TBE 30–80, William A. Cook Australia Pty. Ltd, Brisbane, Australia) was inserted via the left femoral artery (Fig 2). A small endoleak was noted after deployment but was controlled by partial inflation of the SBT oesophageal balloon. The patient returned to ICU with Vancomycin, Timentin and Fluconazole antibiotic cover and his coagulopathy was corrected. A repeat CT angiogram after 24 hours showed no leak after deflation of oesophageal balloon of the SBT. There was significant haematoma within the mediastinum. The SBT was removed the next day without complication. An upper GI endoscopy, five days later revealed a deep oesophageal ulcer at 24 cm with superficial ulceration extending to 29 cm circumferentially. A naso-jejunal feeding tube was inserted at that time.

Subsequently, a percutaneous endoscopic gastrostomy (PEG) was performed. Repeat endoscopy at one month, showed an improvement in the superficial ulceration but the aortic stent was visible through the wall at 24 cm. The patient was maintained on broad spectrum antibiotics delivered via the PEG tube whist discussions were held between him and his family with respect to the management options. However, 51 days after the aortic stent-graft insertion the patient developed a spiking pyrexia and general malaise. Blood cultures were positive for a methicillin-resistant *staphylococcus aureus *(MRSA) and he was recommenced on IV Vancomycin. Repeat CT angiogram revealed no mediastinal gas, fluid levels or significant soft tissue changes to suggest mediastinitis. It was felt the bacteraemia was related to the infected aortic stent-graft.

After multidisciplinary surgical discussion, a plan for definitive treatment was made. Firstly, the patient had a cervical oesophagostomy with the site of division guide by the CT scan to ensure no dissection close to the fistula. A left hemithyroidectomy was needed to allow a tension-free stoma. Two days later, when an aortic allograft became available, the aortoesophageal fistula was repaired. Initially, a left trapezius muscle flap was raised so that this could be brought through the posterior thoracic wall via a window created from partial resection of the fourth and fifth left ribs. After raising the flap, the patient was placed supine and the mediastinum was accessed via a clam-shell incision. We went on cardio-pulmonary bypass with access via left common femoral artery, ascending aorta and right atrium. Retroplegia and left ventricular vent were inserted, the latter via left superior pulmonary vein. The patient was placed into deep hypothermic arrest to 20°C. The aorta was opened from distal arch to 1 cm beyond the distal extent of the endoluminal stent. The descending aorta was cross-clamped and antegrade cerebral perfusion re-established. The stent-graft was removed and sent for microbiological assessment along with involved aorta including the proximal left subclavian artery. The aortic homograft was anastomosed proximally to the isthmus of the arch. The trapezius muscle flap was pulled through from behind. The aortic side branches under-run and the homograft positioned and anastomosed distally. Following re-warming and the patient coming off cardio pulmonary bypass the remaining oesophagus was resected to the level of the azygous vein out of the operative field. Drains were placed in both pleural spaces and in the mediastinum with vacuum suction drains to flap site. Parentral antibiotic cover consisted of Meropenum for two weeks and Vancomycin for four weeks. Cultures from the stent and aortic tissues grew MRSA.

Ten weeks after the aortic repair, the patient was readmitted for reconstitution of the gastrointestinal tract. The colon was mobilised on the ascending branch of the left colic vascular pedicle. During the development of a retrosternal tunnel there was substantial venous bleeding related to adhesion of the right ventricle to the posterior sternum. A median sternotomy was performed and two tears in the right ventricular wall were repaired with bovine pericardium. Because of dense adhesions retrosternally, the use of a retrosternal tunnel was abandoned and a subcutaneous tunnel created. Due to tension from the skin closure on the interposed colon, the colon was returned to the abdomen and the subcutaneous tunnel was stretched overnight with the aid of a half filled litre bag of saline used as a tissue spacer. The following day, the colonic interposition graft was taken to the neck via the subcutaneous tunnel and anastomosed to the proximal oesophageal remnant. There were no complications and he was commenced on oral fluids at day nine after a contrast swallow showed no leak and good passage through the conduit. He was discharged home one week later tolerating normal oral diet. The patient is well with normal activity, 19 months after the original diagnosis of aortoesophageal fistula with minor gastro-oesophageal reflux being his only symptom.

## Discussion

Aortoesophageal fistula (AEF) is a rare and highly dangerous complication of foreign body ingestion. Nandi et al [1], reviewed 2394 cases of impacted oesophageal foreign bodies and two (0.08%) developed an AEF's while Lai et al [2] looked at a consecutive series of 1338 foreign body ingestion and none had an AEF's. Although AEF is a rare complication of foreign body ingestion, foreign bodies are the second leading cause of an AEF's at 19% after thoracic aortic aneurysms at 51% [3].

The long term survival after such a complication is dismal. Excluding our patient, only six cases recorded in the literature with AEF from a foreign body have survived over 12 months, all requiring immediate and aggressive surgical intervention [4-9]. The first case was in 1980 described by Ctercteko and Mok [4], who cross-clamped the descending thoracic aorta and primarily closed the oesophagus and aorta with suture. The most recent case involved emergency endovascular repair with a covered stent as a stabilising measure before definitive surgical treatment [9]. We would like to discuss modern techniques and management strategies we employed in our case, which could optimise the conditions for a favourable outcome in future cases.

Risk factors predicting complications after foreign body ingestion include delayed presentation of more than two days and foreign body impaction [2]. Fish bones have a tendency to migrate, albeit rarely, and have caused cardiac tamponade, a thyroid mass, and been found in the liver, after ingestion [10]. Current guidelines recommend that urgent endoscopic intervention should be performed when a sharp object or disk battery is lodged in the oesophagus [11].

Recognising the warning signs of a potential AEF is important for proper management of these patients. Chiari [12] described a triad of symptoms including mid-thoracic pain, sentinel arterial upper GI haemorrhage and exsanguination after a brief period. These symptoms are more specific in patients with AEF from foreign bodies than from other causes [3]. Often the interval between sentinel haemorrhage and exsanguination is more that 24 hours [3]. This 'window of opportunity' should lead to early transfer of the patient to a centre capable of treating AEF and save valuable time which fortunately happened in our patient.

Initial control of torrential bleeding is challenging. All recent surviving cases had used a Sengstaken-Blakemore tube (SBT) inserted to gain temporary control before going to the operating theatre [5-9]. Endovascular intervention has now become a useful option as shown by our patient and in an earlier reported case by Assink et al [9]. Debate has begun regarding whether endovascular stenting could be used as a definitive procedure or as a short term measure before definitive open surgical treatment. Kato et al [13] successfully treated an AEF secondary to radiation therapy in a patient with oesophageal carcinoma. That case had a low risk of secondary infection compared to our patient. Burks et al [14] reviewed their 5-year experience of bleeding aortoenteric fistulas repaired by endovascular stents and suggested they should be used as a temporary measure before surgery. We feel from our experience, because infection is invariably present after foreign body erosion of the oesophagus, endovascular thoracic aorta stenting should be used as a stop-gap before definitive open surgery.

For the salvage surgery, the second author was guided by literature on managing infected thoracic aorta grafts. It is generally accepted that thorough surgical debridement, antiseptic irrigation and appropriate antibiotic therapy are necessary [15]. The most common pathogens are gram positive cocci (70%) with *staphylococcus aureus *making up 45% of the total [16]. Most centres also use a vascular tissue flap to reduce dead space, the most common being an omental flap [17]. Vogt et al recommended deep hypothermic arrest for surgery on the descending thoracic aorta fistulas[18].

Where grafts are implanted by open surgery, controversy remains regarding whether to completely or partially excise the infected graft and what to replace it with [17]. A case series of 11 patients by Vogt et al [18] showed promising results by using cryopreserved homograft to repair aortoesophageal, aortoenteric and aortobronchial fistulas. Coselli et al [16] also suggested an advantage of homograft in resisting infection after replacing an infected thoracic aorta graft. We felt using a homograft would give the best long term result in view of the resistant organism that was causing the infection.

## Conclusion

Aortoesophageal fistulas secondary to foreign body ingestion are rare but almost always lethal. Chiari's triad of signs and symptoms along with a clear history should alert the treating medical officer of the impending danger. Management initially should include transferring the patient to an appropriate tertiary treatment centre. Aggressive fluid resuscitation and control of an exsanguinating bleed with a Sengstaken-Blakemore tube offers the chance for further treatment. We recommend, if the patient is stable enough for transfer to radiology dept, radiological assessment, with either CT angiogram or arch aortogram and placement of an endovascular stent-graft, can be a further stabilising measure. In the future, endovascular stent-graft with infection resistant material may become the only treatment required but as yet, we advise planned aggressive open surgical treatment as the definitive treatment for long-term results. We believe thorough debridement of necrotic and infected material, utilising a vascular flap to cover the new conduit and use of a homograft, optimise the chance of a successful outcome. This should be done under deep hypothermic arrest.

Unfortunately there is a lack of consensus between centres regarding the treatment of AEF's secondary to foreign bodies. This is almost certainly due to the rarity of the condition and lack of surviving cases in the literature. However, the frequency of long-term survivors is increasing and hopefully remaining questions can be answered with further review and research.

## Consent

Written informed consent was obtained from the patient for publication of this case report and any accompanying images. A copy of the written consent is available for review by the Editor-in-Chief of this journal.

## Competing interests

The authors declare that they have no competing interests.

## Authors' contributions

SK drafted the document and reviewed the literature. PP was joint lead surgeon in the case. PP revised and critically reviewed the document. MO inserted endovascular stent. MO revised and critically reviewed the document. AL revised and critically reviewed the document. MS was joint lead surgeon in the case. MS revised and critically reviewed the document. All authors read and approved the final manuscript.
